# The Effect of Long-Term Second-Generation Antipsychotics Use on the Metabolic Syndrome Parameters in Jordanian Population

**DOI:** 10.3390/medicina55070320

**Published:** 2019-06-28

**Authors:** Osama Abo Alrob, Sayer Alazzam, Karem Alzoubi, Mohammad B. Nusair, Haneen Amawi, Reema Karasneh, Abeer Rababa’h, Mohammad Nammas

**Affiliations:** 1 Faculty of Pharmacy, Yarmouk University, Irbid 22110, Jordan; 2 Faculty of Pharmacy, Jordan University of Science and Technology, Irbid 22110, Jordan; 3 Faculty of Medicine, Yarmouk University, Irbid 22110, Jordan

**Keywords:** metabolic syndrome, second-generation antipsychotics, atypical antipsychotics

## Abstract

*Objectives:* The aim of this study was to determine the incidence of metabolic syndrome in patients treated with second-generation antipsychotics (SGAs). *Methods:* In this retrospective study, we reviewed patients’ electronic medical records (EMRs) of all patients who received one SGA for at least six months, excluding patients who were taking other medications that are associated with significant effect on metabolic syndrome. Relevant clinical information was collected prior to starting the SGA and after six months of continuous use of the same SGA. *Results:* A total of 91 patients were included in the study. The majority of patients (72%) were diagnosed with schizophrenia. After six months of taking the SGA, 44% of patients experienced elevated systolic pressure, 54.9% had elevated triglyceride, and 31.9% had impaired glucose levels (*p* value < 0.05). Prior to initiating SGA therapy, 14.3% of patients had metabolic syndrome, while 37.4% had metabolic syndrome after six months of therapy, and it was more prominent in males compared to female patients (*p* value < 0.05)*. Conclusion:* This study found a strong correlation between SGA use and the appearance of metabolic alterations, such as weight gain, glucose intolerance, and increased triglyceride levels. These findings highlight the importance of assessing metabolic deregulations to minimize SGA associated metabolic abnormalities.

## 1. Introduction

Metabolic syndrome is a phenomenon whereby at least three of five metabolic risk factors coexist, these factors being: elevated blood pressure (systolic blood pressure ≥130 mmHg and/or diastolic blood pressure ≥85 mmHg); elevated fasting glucose (≥110 mg/dL); elevated triglycerides (≥150 mmHg); decreased high density lipoproteins (HDL < 40 mg/dL in men and <50 mg/dL in women); and abdominal obesity (waist circumference ≥102 cm in men and ≥88 cm in women) [1]. Other references consider treatment for dyslipidemia and hypertension and previous diagnosis of type II diabetes to be parameters of metabolic syndrome [2,3]. Accordingly, metabolic syndrome is not a single disease; rather, it is a cluster of several risk factors which may induce other life-threatening diseases [4]. These metabolic abnormalities were previously described in the medical literature using distinctive labels, such as syndrome X, insulin resistance syndrome, and the deadly quartet [5]. Currently, the collective term ’metabolic syndrome’ is utilized universally, with a precise definition of each component [1]. Up to one-third of adults from different ethnic groups around the world can currently be classified as patients with metabolic syndrome, which indicates the high prevalence of this condition worldwide. Also, the prevalence of metabolic syndrome correlates positively with age, with people aged 50 years and older having double the prevalence of metabolic syndrome than the younger population of both males and females [6,7]. Patients with polycystic ovary syndrome, HIV positive status, and hypopituitarism also have a higher prevalence of metabolic syndrome components in comparison to other populations [6]. As previously mentioned, the cluster of metabolic syndrome components may lead to other diseases. Specifically, patients diagnosed with metabolic syndrome have higher levels of inflammatory and prothrombotic markers and are at twice the risk of developing diabetes mellitus (DM) and four times the risk of developing cardiovascular disease (CVD). Both CVD and metabolic syndrome are considered detrimental conditions and are leading causes of morbidity and mortality worldwide [8].

A high prevalence of metabolic syndrome factors among psychiatric patients has been reported in several studies. In general, psychiatric patients have been found to have a twofold higher prevalence of DM, hypertension, obesity, and dyslipidemia compared to the general population [9]. Psychiatric patients also have a higher prevalence of cardiovascular lethal events. For psychiatric patients, CVDs are the second leading cause of death after suicide [10]. Metabolic syndrome may be viewed to be a link between CVD and psychiatric diseases [11]. The increased risk of metabolic syndrome among psychiatric patients is in part seen to be a result of the effect of psychiatric diseases, such as schizophrenia and bipolar disorder, on molecular pathways. On the other hand, strong evidence suggests the presence of a link between the development of metabolic syndrome and the chronic use of antipsychotic treatment. Antipsychotic medications are the primary choice of treatment for patients with psychiatric disorders. First-generation antipsychotics (FGAs) were the first antipsychotics to be released to the market; however, FGAs were found to be associated with adverse neurological side effects, such as extrapyramidal side effects and tardive dyskinesia [12]. As a result, second-generation antipsychotics (SGAs), which promised a reduced risk of neurological side effects, were introduced to the market. Despite this, some SGAs have been found to be associated with detrimental metabolic side effects [11].

SGAs may induce cardiometabolic and endocrine side effects, which can be particularly exaggerated in children [13]. The influence of antipsychotic drugs on metabolic syndrome may be observed through changes to the waist circumference, triglycerides levels, and glucose control [14,15]. Blood pressure and serum HDL levels may also be affected but at lower levels. Weight gain becomes markedly evident during the early stages of treatment, especially in patients with low body mass index (BMI) [15]. The suggested molecular mechanisms by which antipsychotic medications induce metabolic changes are multifactorial. Signaling pathways involved in the regulation of histamine, dopamine, neuropeptides, adrenergic and muscarinic receptors, and glucose metabolism are all affected [16]. Further, risk factors, such as modifiable sedentary lifestyle, can increase patient susceptibility to the metabolic effects of antipsychotic medications [17].

Genetic variations, including several single nucleotide polymorphisms (SNPs), have also been identified as non-modifiable risk factors [18]. In support of this, evidence suggests that MTHFR polymorphism may induce metabolic disturbances in patients treated with SGAs [19,20]. Different antipsychotic drugs have different metabolic profiles, with some compounds being more likely to lead to the development of metabolic syndrome than others [21]. For example, olanzapine has been shown to have the greatest effect on waist circumference and triglycerides compared to other antipsychotics, such as quetiapine and ziprasidone [22]. These variations are primarily due to differences in pharmacological receptors, including M_3_, central 5-HT2C, and D2 receptors. Due to this reported evidence about the impact of antipsychotic drugs on metabolic syndrome parameters, several agencies and associations, such as the American Diabetes Association and the American Psychiatric Association, have released reports (ADA/APA Consensus paper) to bring attention to the metabolic consequences of the chronic use of SGAs, including consequences, such as the possible development of hyperglycemia, hypertriglyceridemia, and weight gain. These agencies also recommend the close monitoring of weight, blood sugar, and triglycerides levels during the chronic use of these medications [23]. In Jordan, the prevalence of metabolic syndrome is alarming, and evidence suggests that the prevalence has been gradually increasing over the years [24]. Previous results in Jordan suggest that the prevalence of metabolic syndrome ranged between 51–65% [24,25]. However, the prevalence of metabolic side effects due to SGAs has yet to be evaluated. Therefore, we are eager to test the central hypothesis of this study, which suggests the presence of a high prevalence of metabolic side effects among patients receiving antipsychotic medications in Jordan.

## 2. Methods

### 2.1. Study Design

This study is a retrospective study which examined the effect of SGAs on the risk factors of metabolic syndrome. The electronic medical records (EMRs) of patients at King Abdullah University Hospital (KAUH) were reviewed. Approval to conduct this study was obtained from Jordan University of Science and Technology and the Institutional Review Board (IRB) at KAUH. This study was conducted by an ethics approval obtained on 1 March 2018, reference code 9/113/2018.

### 2.2. Study Population

The inclusion criteria for patients were: (1) adults (defined as aged 18 years and older), (2) have been taking one SGA for at least six months, and (3) have electronic documentation for their follow-up visits and laboratory tests. We excluded: (1) patients who were taking other medications associated with having a significant effect on metabolic syndrome clinical indicators (i.e., Lithium; corticosteroids; anti-hypertensive medications; and lipid and glucose lowering agents), (2) patients taking more than one antipsychotic, and (3) patients with more than 20% of missing values.

### 2.3. Data Collection and Measures

Patients were identified using the electronic records at KAUH. Patient demographics and any relevant clinical information (using both EMRs and follow-up charts) were collected. Laboratory test results prior to the initiation of the SGA (time 1) and after six months of continuous use of the same SGA (time 2) were also collected. Laboratory tests included: HDL, triglycerides, fasting blood glucose, and blood pressure. Waist circumference measures were not available; therefore, this parameter was not assessed. However, BMI was assessed as an alternative [26]. According to the World Health Organization (WHO), a BMI of 30 kg/m^2^ and above indicates central obesity, which is one of the components of metabolic syndrome [27]. In this present study, due to the lack of waist circumference measures, BMI will be utilized as a predictor for central obesity.

### 2.4. Statistical Analysis

Descriptive measures included frequencies and percentages for all categorical variables and mean  ±  standard deviation for continuous measures, respectively. Two-tailed t-test was used to analyze continuous variables, and Chi square test was used for categorical variables. All statistical analyses were performed using the Statistical Package for the Social Sciences (SPSS) version 25.

## 3. Results

A total of 91 patient records met the inclusion and exclusion criteria. The mean age was 41.4 (SD 16.13) years, and 51.6% of the records were for male patients (Table 1). The majority of the patients had been diagnosed with schizophrenia (72%), whilst the remaining 38% were bipolar patients. Thirty-five patients were on olanzapine (38.5%), 16 on risperidone (17.6%), 31 on quetiapine (34%), 8 on aripiprazole (8.8%), and one on clozapine (1.1%). Overall, the mean values for metabolic syndrome factors prior to the initiation of SGA intake (time 1) were below the Adult Treatment Panel (ATP) III criteria (Table 2). The mean values for triglyceride, blood pressure (both systolic and diastolic), fasting glucose, and BMI showed to have increased significantly after six months of SGA intake (time 2; Table 2). At time 2, the mean values for fasting glucose and triglyceride among male patients were found to be above 110 and 150 mg/dL, respectively (Table 2). As for female patients, they did not experienced a significant increase in blood pressure (both systolic and diastolic), and the total mean value for fasting glucose remained within the normal range (Table 2).

At baseline, 44% of patients had HDL cholesterol readings that met the ATP III criteria for metabolic syndrome. However, less than 20% of the patients met any of the other metabolic syndrome criteria at time 1 (Table 3). At time 2, more than 40% of the patients had systolic blood pressure and triglyceride readings that met the ATP III criteria for metabolic syndrome (Table 3). Moreover, the number of patients with BMI ≥30 kg/m^2^ had nearly doubled after six months of the initiation of SGA. Prior to initiating SGA, 13 (14.3%) patients, seven (53.8%) of whom were female (Table 4), had metabolic syndrome (three or more ATP III criteria). At time 2, 35 patients (37.4%) had developed metabolic syndrome (*p* value <0.05; Table 4). Further, male patients had seen a significant development of four metabolic syndrome criteria (*p* value <0.05; Table 4).

## 4. Discussion

Psychiatric patients who take antipsychotics are known to be at a high risk of developing metabolic syndrome and cardiovascular associated diseases [29]. However, the clinical metabolic effects of SGAs in the Jordanian population was never addressed. The current study provides new insights on the detrimental effects of the long-term use of SGAs on metabolic syndrome. The study results indicate a positive correlation between the chronic use of SGAs and the development of metabolic syndrome in both genders. Antipsychotics can cause direct damage to β-cells, which leads to decreased insulin secretion [30,31,32]. Moreover, previous studies have reported that SGAs inhibit Akt activity, which, in turn, inhibits the insulin signaling pathway in insulin sensitive cells (e.g., muscle cells, hepatocytes, etc.). Eventually, this leads to insulin resistance [30,33].

**Figure 1:**
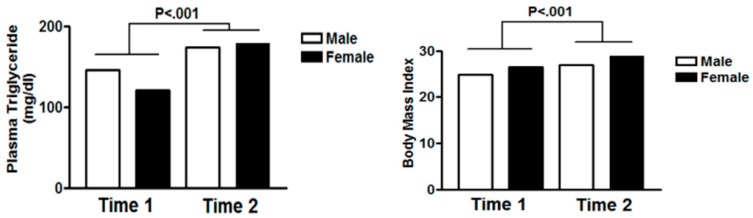
Plasma Triglyceride and BMI variation across genders six months post-SGAs therapy.

In a recent cross-sectional study, prediabetes and metabolic abnormalities were found to be highly prevalent among schizophrenic patients taking clozapine or olanzapine [34]. Interestingly, previous reports have found that patients who are on clozapine or olanzapine are at a high risk of developing hyperlipidemia [35]. The strong correlation between the long-term use of clozapine or olanzapine and lipid deregulation is mainly attributed to weight gain, dietary changes, and glucose intolerance. In agreement, our study results indicate a significant increase in the plasma levels of triglycerides after six months of SGA treatment (Figure 1). Moreover, olanzapine has been found to deregulate hepatic lipid metabolism and exacerbate atherosclerosis in apolipoprotein E-null (apoE^-/-^) mice. Given the fact that dyslipidemia and atherosclerosis are major risk factors for CVDs, this may explain in part the high prevalence of CVDs in psychotic patients on SGAs [36]. Furthermore, antipsychotic weight gain has been found to be a common adverse effect of chronic treatment [37]. SGA-induced obesity can cause insulin resistance accompanied by high levels of free fatty acid and inflammation [30,38]. Our results showed that the average weight of patients from both genders had increased after six months of treatment with SGAs, as indicated by the significant increase in BMI (Figure 1). Olanzapine significantly decreases the appetite-stimulating hormone ghrelin, which leads to obesity in schizophrenic patients [39]. In agreement, weight gain has recently been reported to be particularly prevalent among Chinese patients on SGAs, suggesting that many genes may be affected by the long-term use of antipsychotic drugs. Specifically, HTR2C was found to be the most strongly associated gene with antipsychotic obesity in the Chinese population [40]. Moreover, evidence shows that more than 20% of Turkish patients on SGAs experienced weight gain [41], which sheds light on the importance of weight control strategies for psychiatric patients on SGAs.

Intriguingly, our current study revealed that the chronic use of SGAs increases both systolic and diastolic blood pressure in male patients only. The female patients seemed to have more stable readings of blood pressure, despite the long-term use of SGAs. Parks et al. reported an acute decrease in blood pressure in patients three days post-initiation of SGA [42]. Moreover, mean systolic blood pressures were found to have had slightly decreased after 12 months of SGA use [43]. A large body of evidence suggests that orthostatic hypotension is one of the most common side effects of antipsychotic drugs [44]. In the current study, however, systolic and diastolic blood pressures were found to be significantly high in patients taking SGAs. This may be explained as being a result of arterial stiffness [45]. Collectively, results from our current study and from other studies indicate that acute and chronic changes in blood pressure may accompany the use of different SGAs; however, these findings require further investigation by prospective studies on various patient populations.

Gender based differences in the effects of the long-term use of antipsychotics were noticed in this study; specifically, male patients experienced a significant development of four metabolic syndrome criteria. Genetic- and gender-based differences in the effects of SGAs have previously been reported [46]. Plasma concentration of antipsychotic drugs has shown to produce varied results based on age and gender. For example, olanzapine plasma concentration was found to be higher in older patients compared to younger patients and lower in blood concentration in males compared to females [47]. Furthermore, clozapine treated schizophrenic men had significantly higher odds for most of the metabolic syndrome criteria compared to treated female patients [48].

### Limitations

This study is a retrospective study with few limitations. This study design has two possible threats: single group threat (i.e., lack of comparison or control group) and historical threat (i.e., other factors or interventions possibly influencing the outcomes) [49]. Moreover, the lack of waist circumference measurements in the EMRs limited the accurate reporting of metabolic syndrome prevalence among the study population. Furthermore, accessibility to patient profiles was limited. Some patient profiles were classified and required special access permission; this was to protect the identity of these patients due to the stigma associated with psychiatric disorders.

## 5. Conclusions

**Figure 2:**
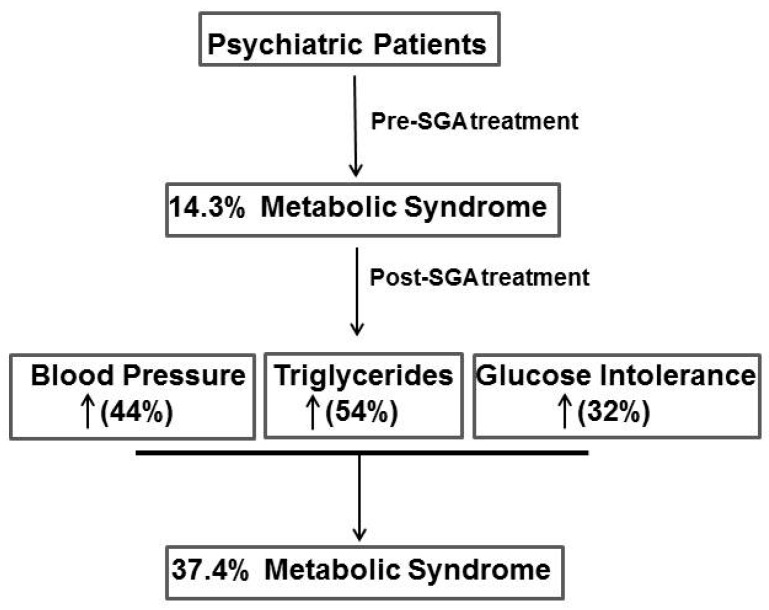
Diagram that illustrates the effect of SGA medications use on metabolic syndrome parameters in the study population.

In conclusion, our study has revealed a strong correlation between the use of SGAs and the development of metabolic alterations, such as weight gain, glucose intolerance, and increased triglyceride levels (Figure 2). Furthermore, there were differences between males and females with regard to the side effects of the long-term use of SGAs. Our findings highlight the importance of assessing metabolic deregulations in order to minimize SGA-related metabolic abnormalities.

## Figures and Tables

**Table 1:** Comparison of second-generation antipsychotics (SGAs)on different metabolic syndrome parameters.

	Weight Gain	Hypercholesterolemia	Blood Pressure	Hyperglycemia
**Olanzapine**	+++	+++	+/−	+++
**Clozapine**	+++	+++	-	+++
**Risperidone**	++	+	-	+
**Quetiapine**	++	++	-	++
**Aripiprazole**	+/0	+/0	-	+/0
Ziprasidone*	+/0	+/0	-	+/0

* None of this study population was taking ziprasidone. Table adjusted from Ref. [28] © Copyright Frontline Medical Communications. All rights reserved. Reproduced with permission.

**Table 2:** Sociodemographic and medical characteristics of study participants (*n* = 91).

Variable		*N* (%)
Gender	Male	47 (51.6%)
	Female	44 (48.4%)
Marital Status *	Single	31 (37.3%)
	Married	40 (48.2%)
	Divorced/Widowed	12 (14.5%)
Living Status *	With family/partner	79 (91.8%)
	Alone	7 (8.2%)
Education Level *	Primary education	18 (22.2%)
	Secondary education	20 (24.7%)
	Post-secondary education	43 (53.1%)
Employment Status	Employed	47.2 (47.2%)
	Unemployed	47 (52.8%)
Age mean (SD)		41.4 (16.13)

* These attributes contain missing values.

**Table 3:** Mean values of metabolic syndrome criteria pre- and post-six months of SGAs therapy.

Metabolic Syndrome Factor	ATP III Criteria*	Time 1 Mean (SD)			Time 2 Mean (SD)			*p* Value		
		Total	Male	Female	Total	Male	Female	Total	Male	Female
Systolic Blood Pressure	*≥130 mmHg*	121.6 (16.34)	120.1 (16.62)	123.2 (16.07)	125.7 (11.95)	126.8 (9.70)	124.5 (13.98)	0.045	0.014	0.660
Diastolic Blood Pressure	*≥85 mmHg*	75.3 (9.66)	73.6 (9.96)	77.2 (9.07)	79.7 (9.63)	80.3 (10.14)	79.0 (9.12)	0.001	<0.001	0.268
Triglyceride	*≥150 mg/dL*	133.6 (85.87)	146.0 (104.77)	120.49 (57.84)	176.1 (100.49)	173.3 (103.91)	179.1 (97.80)	<0.001	0.001	<0.001
High density lipoproteins (HDL) Cholesterol	*<40 mg/dL for men <50 mg/dL for women*	45.9 (21.37)	45.3 (24.82)	46.6 (17.18)	43.4 (13.49)	42.4 (14.05)	44.42 (12.93)	0.274	0.280	0.280
Fasting Glucose	*≥110 mg/dL*	96.8 (28.16)	99.6 (35.98)	93.7 (16.03)	112.66 (49.79)	116.9 (62.4)	108.2 (31.32)	0.001	0.031	0.003
Body Mass Index (BMI)	-	25.6 (4.38)	24.8 (3.05)	26.5 (5.34)	27.9 (5.88)	26.9 (3.68)	28.9 (7.43)	<0.001	<0.001	0.009

* ATPIII: Adult Treatment Panel III [4].

**Table 4:** Frequency of patients meeting metabolic syndrome criteria pre- and post- six months of SGAs therapy.

Metabolic Syndrome Factor	ATP III* Criteria for Metabolic Syndrome	Time 1 *N* (%)	Time 2 *N* (%)	*p* Value
Systolic Blood Pressure	≥130 mmHg	14 (15.4%)	40 (44.0%)	<0.001
Diastolic Blood Pressure	≥85 mmHg	18 (19.8%)	26 (28.6%)	0.113
Triglyceride	≥150 mg/dL	23 (25.3%)	50 (54.9%)	<0.001
HDL cholesterol	<40 mg/dL for men	40 (44.0%)	38 (41.8%)	0.440
	< 50 mg/dL for women			
Fasting glucose	≥110 mg/dL	12 (13.2%)	29 (31.9%)	<0.001
BMI ≥30kg/m^2^	-	12 (13.3%)	21 (23.3%)	0.061

* ATPIII: Adult Treatment Panel III [4].
